# Successful closure of a duodenal stump leak with an over-the-scope clip using double-balloon endoscope

**DOI:** 10.1055/a-2447-8139

**Published:** 2024-11-22

**Authors:** Toru Kuwano, Shunji Shimaoka, Hirotake Kusumoto, Hideyuki Kishita, Tsutomu Sakiyama, Shinya Yamamoto, Yu Horikawa

**Affiliations:** 1 73609 Department of Gastroenterology, Nanpuh Hospital, Kagoshima, Japan

A 62-year-old man with a history of distal gastrectomy and Billroth I reconstruction for gastric cancer was diagnosed with early gastric cancer in the remnant stomach, necessitating endoscopic submucosal dissection. On postoperative day 1, the patient experienced sudden-onset abdominal pain. Computed tomography (CT) demonstrated free air and mediastinitis, indicating a delayed perforation. Total gastrectomy with Roux-en-Y reconstruction, irrigation, and drainage was subsequently performed.

On postoperative day 5, bloody discharge was observed coming from the drain. On postoperative day 6, double-balloon endoscopy revealed two duodenal stump leaks measuring approximately 4 mm and 3 mm, respectively, with brisk bleeding in one of them ( Fig. 1 , Fig. 2 ). Repeat CT identified the gastroduodenal artery as the source of bleeding ( Fig. 3 ), and arteriogram coil embolization successfully achieved hemostasis. Considering that fistulas could cause intra-abdominal rebleeding or intra-abdominal abscesses, closure using over-the-scope (OTS) clips was planned. However, the diameter of the double-balloon endoscope tip was smaller than that of compatible OTS clips, complicating the mounting process. To address this, vinyl tape was wrapped around the scope tip several times to increase its outer diameter, enabling OTS clip mounting onto the scope ( Fig. 4 , Video 1 ). This facilitated successful fistula closure using OTS clips ( Fig. 5 ), significantly improving the patient’s condition without complications or recurrent leaks.

**Fig. 1:**
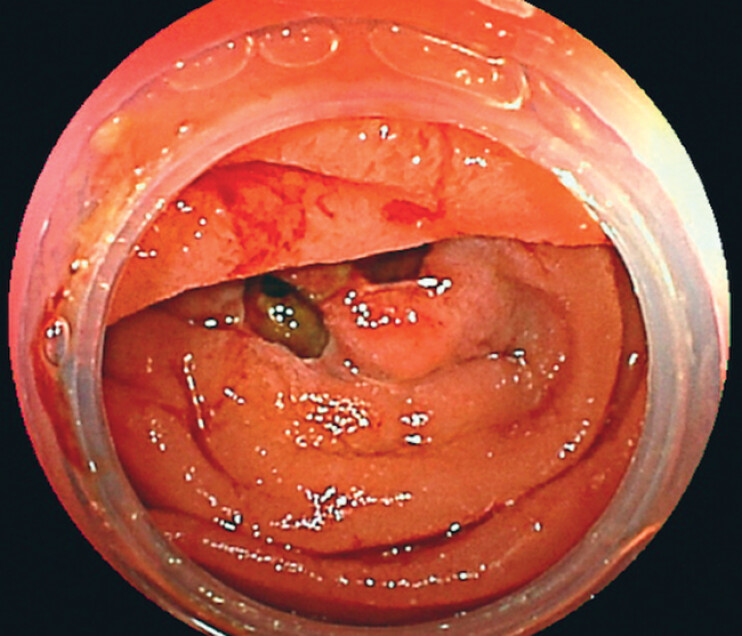
Endoscopic image demonstrating two duodenal stump fistulas measuring approximately 4 mm and 3 mm in diameter, respectively.

**Fig. 2:**
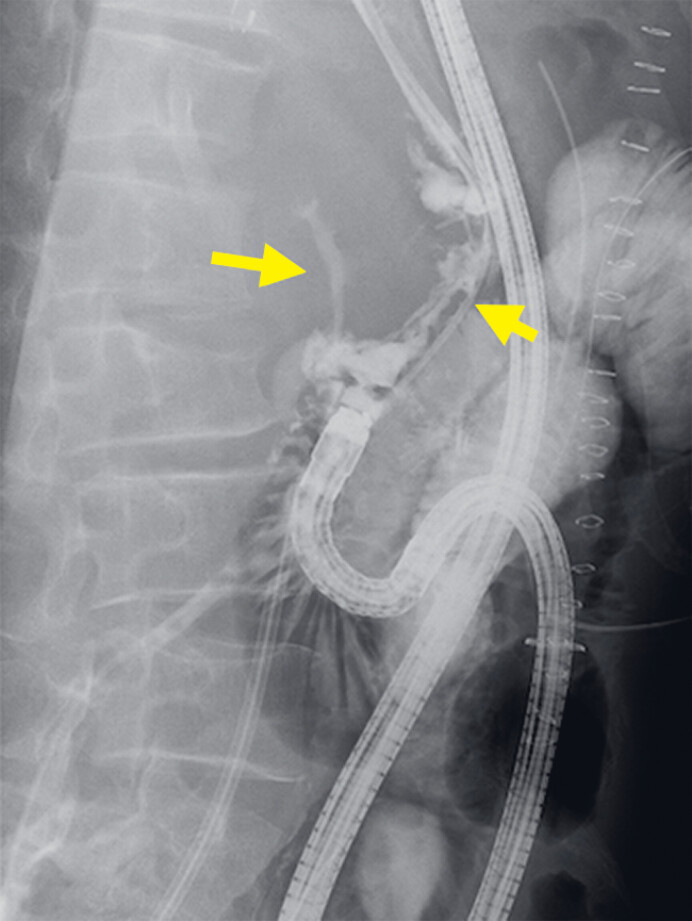
Fluoroscopic endoscopy showing contrast agent leaking outside the duodenal stump (yellow arrowheads).

**Fig. 3:**
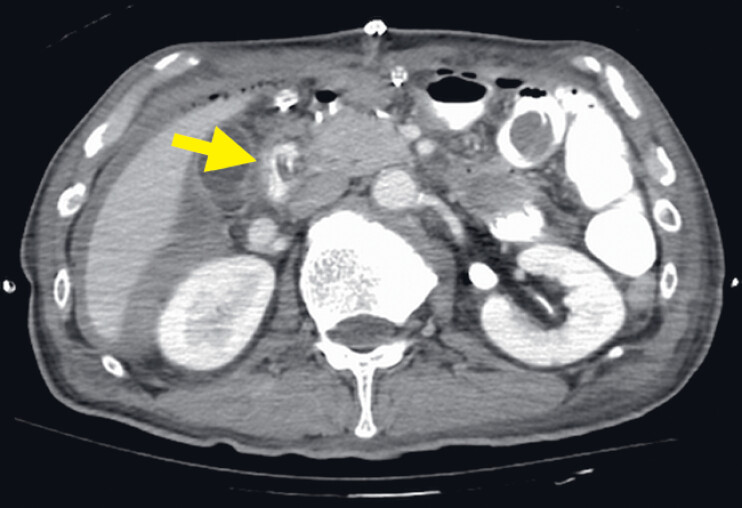
Contrast-enhanced computed tomography showing extravasation from the gastroduodenal artery (yellow arrowheads).

**Fig. 4:**
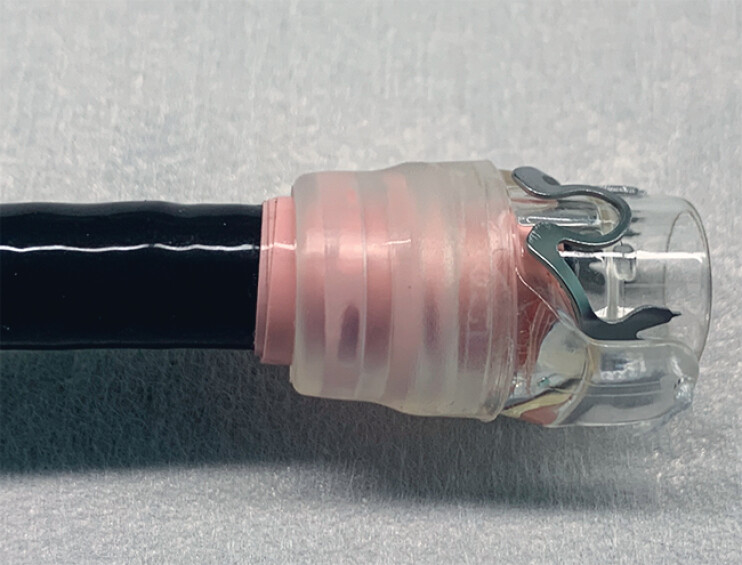
The over-the-scope clip was mounted onto the tip of the double-balloon endoscope.

**Fig. 5:**
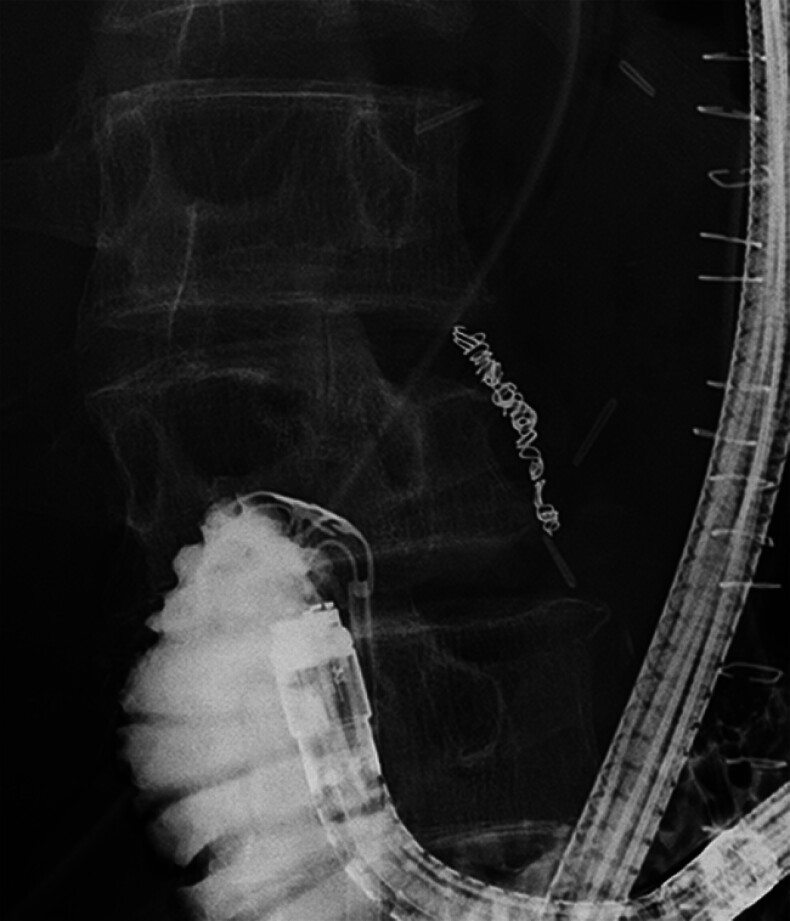
Successful closure of the duodenal stump leak was achieved.

We wrapped vinyl tape around the scope tip several times to increase the outer diameter, making it possible to mount the over-the-scope clip onto the double-balloon endoscope.Video 1

OTS clips are a novel endoscopic modality used to close gastrointestinal defects 1 2 . Duodenal stump leakage is a serious post-gastrectomy complication 3 , and endoscopic treatment is technically challenging in patients who have undergone Roux-en-Y reconstruction 4 . This case highlights the significant therapeutic potential of OTS clips with a double-balloon endoscope for closing fistulas, even in complex postoperative reconstructions.

Endoscopy_UCTN_Code_TTT_1AO_2AI

Endoscopy_UCTN_Code_TTT_1AP_2AD

## Notes

Endoscopy E-Videos https://eref.thieme.de/e-videos
*E-Videos* is an open access online section of the journal *Endoscopy* , reporting on interesting cases and new techniques in gastroenterological endoscopy. All papers include a high-quality video and are published with a Creative Commons CC-BY license. Endoscopy E-Videos qualify for HINARI discounts and waivers and eligibility is automatically checked during the submission process. We grant 100% waivers to articles whose corresponding authors are based in Group A countries and 50% waivers to those who are based in Group B countries as classified by Research4Life (see: https://www.research4life.org/access/eligibility/ ). This section has its own submission website at https://mc.manuscriptcentral.com/e-videos .
